# Descending Mediastinitis in a Child: Diagnostic Challenges and a Different Treatment Approach

**DOI:** 10.3390/clinpract11030066

**Published:** 2021-08-09

**Authors:** Joana Lira, Joana Santos, Mariana Capela, Joana Rodrigues, Otília Cunha, Isabel Carvalho

**Affiliations:** Pediatrics and Neonatology Department, Centro Hospitalar Vila Nova de Gaia/Espinho, 4434 Vila Nova de Gaia, Portugal; joana_csantos@hotmail.com (J.S.); mariana.capela@chvng.min-saude.pt (M.C.); joana.rodrigues@chvng.min-saude.pt (J.R.); otilia.cunha@chvng.min-saude.pt (O.C.); maria.isabel.carvalho@chvng.min-saude.pt (I.C.)

**Keywords:** group a streptococcus, deep cervical infection, ultrasound monitoring

## Abstract

In children, spontaneous mediastinitis is a rare, severe and commonly misdiagnosed disease. Although standard of care treatment frequently involves surgery, we report a case of mediastinitis in a five-year-old child, successfully treated with 4 weeks of intravenous antibiotics. Ultrasound imaging was used to monitor patient response to conservative treatment while reducing radiation exposure.

## 1. Introduction

Mediastinitis is a severe inflammation of the mediastinal connective tissue associated with high morbidity and mortality. In childhood, as in adults, it is a rare disease, most frequently arising as an infectious complication of transthoracic procedures or esophageal perforations [1]. Alternatively, mediastinitis may also arise from head or neck infections extending to the mediastinum (descending mediastinitis), which, in the antibiotic era, is even rarer [1,2,3]. Clinical presentation includes fever and mostly respiratory symptoms, such as tachypnea, retractions and cyanosis [4,5]. Besides long-term intravenous antibiotics, treatment usually includes surgical drainage [6,7]. There is a lack of references advocating only medical management of the disease. To our knowledge, there is only one previously reported case in a pediatric case series [8]. In this report, the authors propose an antibiotic only conservative approach as a treatment option to consider in clinically stable pediatric patients and highlight the possibility of using ultrasound (US) imaging to monitor patient’s response.

## 2. Case Report

A five-year-old girl, otherwise healthy, presented to the Emergency Department (ED) with low-grade fever and a sore throat. She was discharged with symptomatic treatment but returned 24 h later due to higher and more frequent fever (max 40 °C), associated with thoracoabdominal pain and anorexia.

On physical examination the patient had fever (38 °C), tachypnea (45 breaths/min), a limitation of neck movements, trismus, non-purulent pharyngitis, chest indrawing and bibasilar crackles on pulmonary auscultation. Laboratory results showed leukocytosis (31,490 /mm^3^), neutrophilia (27,210 /mm^3^), and elevated C-reactive protein (34.03 mg/dL). Rapid antigen detection test for Group A Streptococcus (GAS) was negative.

**Figure 1:**
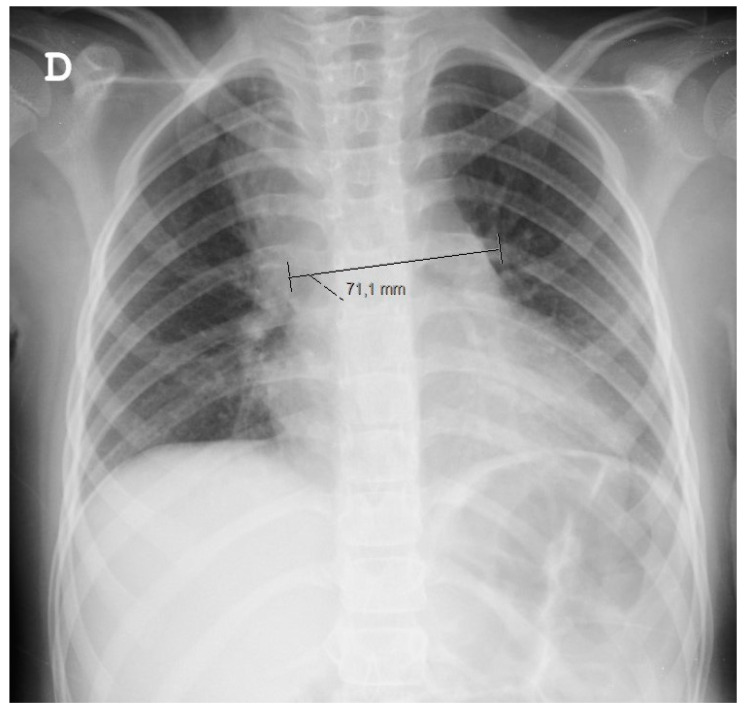
Chest radiograph. D stands for “direita”—right in Portuguese.

Since a chest X-ray (Figure 1) raised doubts about a superior right lobe condensation and a widened mediastinum, she was started on ampicillin and underwent a thoracic CT scan. The CT scan confirmed a widened mediastinum as a result of three fluid collections (the largest having 40 × 14 × 27 mm), topographically related to lymphadenopathies, which were associated with adjacent mediastinal tissue densification. Such findings prompted treatment conversion to large spectrum IV antibiotics (ceftriaxone and vancomycin).

A needle aspiratory biopsy, a peripheral blood immunophenotyping and tumor markers (alpha fetoprotein and human chorionic gonadotropin), excluded a malignant cause.

**Figure 2:**
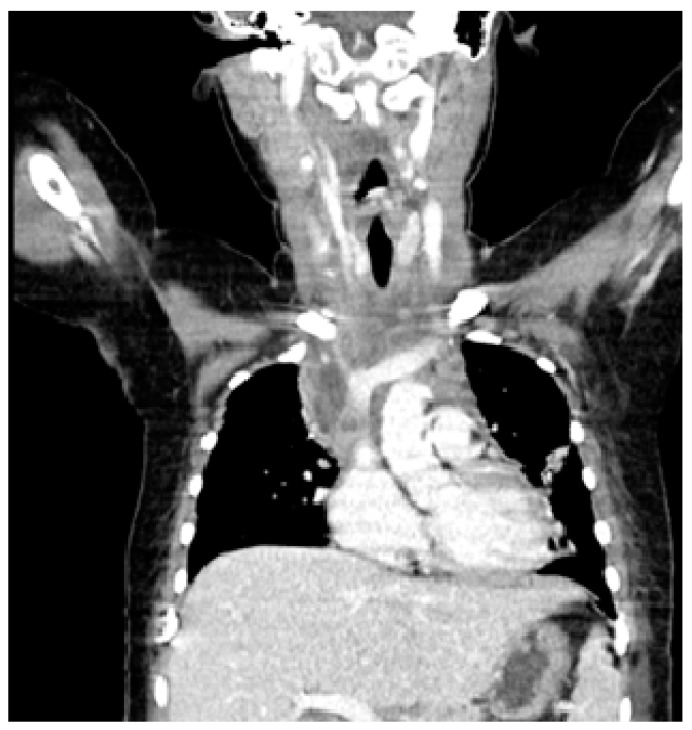
Neck and Chest CT Scan.

On the fourth day of admission a GAS was isolated from a blood culture and IV clindamycin was added to her treatment. When repeated to include the cervical area, in addition to the known mediastinal lesions, a CT scan revealed edema and densification of the retropharyngeal space, suggesting a descending mediastinits extending from a cervical infection (Figure 2). There was no air suggestive of an esophageal perforation.

**Figure 3:**
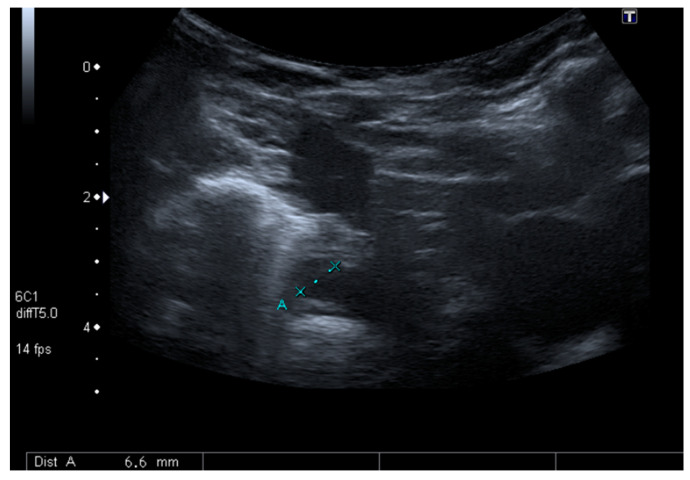
Follow up mediastinal ultrasound.

Although surgical drainage was considered, a follow-up CT scan showed a sustained decrease of the mediastinal lesions with antibiotics only. For this reason, the child was maintained under close surveillance with serial US (Figure 3). She maintained fever until the 6th day of antibiotics, after which a sustained clinical and laboratorial improvement was achieved. At the end of 4 weeks of IV antibiotics, the child was discharged without any symptoms and with complete resolution of the mediastinal lesions.

## 3. Discussion

Acute mediastinitis presents suddenly without any specific signs. Suspicion must be high in order to make a prompt diagnosis even in the absence of an obvious trigger [3,6]. In this case, the child presented to the ED, seriously ill, with a wide range of symptoms. Trismus and decreased range of the neck movement, in addition to radiologic findings of a widened mediastinum, must raise suspicion of a deep cervical infection that can, through the retropharyngeal space, spread as a descending mediastinitis [5].

As for the identified agent, GAS is an aggressive bacteria, responsible for serious soft tissue infections, that has been specifically associated with descending mediastinitis [5,6,9,10].

Lastly, existing literature from adult and pediatric patients highlights the importance of a prompt drainage. However, by the time we made the diagnosis, on day 4, there was no cervical collection and the mediastinal lesions were resolving, along with a sustained clinical and laboratorial improvement. The decision for a watchful waiting approach turned out to be successful. Since US was easily available and proved accurate, without having to subject the child to more radiation, we decided to use it to closely monitor the mediastinal lesions.

The authors thereby emphasize that, although CT scan is the gold standard for the diagnosis and control of acute mediastinitis, US imaging must be considered as an alternative radiological tool, reducing radiation exposure, whilst allowing clinicians to avoid surgical drainage in selected stable cases.
